# The Nature of Reality: Human Stress Recovery during Exposure to Biodiverse, Multisensory Virtual Environments

**DOI:** 10.3390/ijerph17010056

**Published:** 2019-12-19

**Authors:** Morgan Faith Schebella, Delene Weber, Lisa Schultz, Philip Weinstein

**Affiliations:** 1 Natural and Built Environments Research Centre, University of South Australia, Mawson Lakes, SA 5095, Australia; delene.weber@unisa.edu.au; 2 School of Information Technology and Mathematical Sciences, University of South Australia, Adelaide 5000, Australia; lisa.schultz@unisa.edu.au; 3 School of Biological Sciences, University of Adelaide, Adelaide 5000, Australia; Philip.weinstein@adelaide.edu.au

**Keywords:** biodiversity, species richness, virtual reality, stress, experiment, well-being

## Abstract

Immersive virtual environments (IVEs) were used to test the effects of biodiversity on recovery from induced stress. Three natural environments and one urban environment were used to represent ordinal levels of biodiversity (none, low, moderate, and high). The four IVEs comprised visual, auditory, and olfactory stimuli. An additional high biodiversity IVE without auditory or olfactory stimuli was also included to study the effects of multisensory stimulation per se on recovery from stress and perceptions of biodiversity. Following stress induction via a novel IVE Trier Social Stress Test (TSST-IVE), heart rate and five self-reported well-being measures were used to assess participants’ recovery after immersion in one of the five IVEs. The results showed consistent well-being responses across both self-reported and physiological measures, suggesting biodiversity does directly affect human well-being. However, the relationship was not linear. For most measures of well-being, stress recovery was least effective in the urban IVE, consistent with past research. The low biodiversity IVE elicited the greatest improvement in all well-being measures except self-reported calmness. One could speculate that the landscape features of the least biodiverse IVE may elicit subconscious preferences toward savanna-like landscapes, as suggested by previous studies. The IVE depicting a moderate level of biodiversity was the least restorative of the natural environments. A multisensory experience was associated with better recovery in all measures of well-being than a visual-only experience, and perceptions of landscape components significantly differed between two identical nature scenes when auditory and olfactory stimuli were removed. Nuances in the data and implications of the findings are discussed. The results signal a need for caution and question the assumption that cultural ecosystem services align with positive outcomes for biodiversity conservation.

## 1. Introduction

The world might be a better place were the following not true: that which is good for people is not always good for nature. Although a plethora of research supporting a link between nature and human well-being may, superficially, be suggestive of a mutually beneficial relationship, the vast majority of studies have compared the benefits derived from broadly defined “natural” and “built” environments, with far fewer studies having examined variations in natural settings or given consideration to ecological quality or ecosystem functioning (for review, see Reference [1]). In fact, most studies in this field have failed to describe the natural environment at all [2]. Despite global initiatives such as “Healthy Parks, Healthy People”, we find that, comparatively, very little attention has been paid concurrently to the health and resilience of green spaces and to the benefits those environments provide to humanity. Indeed, some researchers are concerned that green space has become a commodity, whereby we carefully select the attributes we believe it should contain based on the benefits we attain from them and risk creating a “watered-down, biodiversity poor version of nature with compromised ecosystem services” [3] (p. 3). Human-induced change to the natural environment has resulted in unprecedented rates of species loss, estimated to be 1000 times the background or prehuman rate [4]. This ongoing decline in biodiversity is expected to carry a multitude of repercussions for our health and well-being [5], largely as a consequence of the declining ability of natural environments to provide provisioning (e.g., food), supporting (e.g., nutrient cycling), regulating (e.g., water purification), and cultural (e.g., spiritual connection to nature) ecosystem services [6]. 

### 1.1. Psychological Benefits as an Ecosystem Service

The psychological well-being benefits gained from natural environments are frequently cited as a cultural ecosystem service, e.g., References [6,7]. An important aspect of the widely adopted “ecosystem services” approach to conservation—which focuses on the human benefits derived from nature—is that, although it differs from a strict “biodiversity conservation” approach, in theory, it will still result in positive outcomes for biodiversity whilst also being accessible to a much broader audience who might not necessarily be concerned with species protection [8]. The concern, however, is that if psychological benefits are shown to be derived from depauperate environments, then an “ecosystem services” approach does not serve biodiversity conservation. In such situations, it is vital we acknowledge that the two approaches are unlikely to result in the same outcomes, “so that increasingly scarce resources for biodiversity conservation may be used to target those elements of biodiversity that may not be conserved otherwise” [8] (p. 7).

It is therefore crucial to determine if greater levels of psychological benefit are associated with greater biodiversity per se. Many well-being studies have focused on “parkland with scattered shrubs and trees” [3] (p. 3), and landscape preference research frequently demonstrates that these savanna-like settings are preferred by people, e.g., References [9,10]. Unfortunately, unlike a natural savanna, which may sustain high levels of biodiversity, people’s preferences for modified “savanna-like” settings is associated with the clearing of understory vegetation and presents “a significant obstacle for those seeking to protect biological diversity within ecosystems” [11]. Consequently, it cannot be assumed that classifying nature-derived psychological benefits as a cultural ecosystem service lends itself to biodiversity conservation. Few studies have explored the relationship between biodiversity and psychological benefits directly, and those that have, have produced inconsistent findings. Furthermore, only one experimental study has tested the effects of biodiverse environments on psychological well-being (i.e., Johansson et al. [12]; however, Cracknell et al. [13] also explored this using marine animals in an aquarium setting). The present study seeks to contribute to this topic by testing whether biodiversity influences recovery from induced stress, using an innovative, multisensory Immersive Virtual Environment (IVE) laboratory experiment.

### 1.2. Does Biodiversity Affect Psychological Well-Being?

In their 2010 review, Jorgensen and Gobster noted a dearth of research examining the psychological benefits associated with biodiversity, with only one study having been conducted at that point in time [14]. Recent reviews show that interest in the topic has grown over the past decade [15,16]; however, the results of this research are far from conclusive, and “the topic does not appear to have been addressed in a comprehensive and systematic manner” [16] (p. 5).

Fuller et al. [14] first identified a positive association between self-reported psychological benefits and plant, bird, and habitat diversity and further determined that participants were able to detect plant species richness fairly accurately. In contrast, Dallimer et al. [17] found no consistent relationship between plant species richness and psychological benefits. They found that benefits increased with perceived biodiversity; however, respondents’ perceptions were markedly inaccurate. Further studies have highlighted the inability of laypeople to perceive differences in biodiversity [18,19]. More recently, Carrus et al. [20] found that perceived restorativeness varied amongst four distinct types of green space, ranging from an urban square to a protected peri-urban reserve. These settings reflected differences in biodiversity; however, they also likely differed in other attributes (e.g., size and naturalness), and the researchers did not assess biodiversity perceptions. Van den Berg et al. [21] found no significant differences in perceived restorativeness between three natural conditions using an experimental design to examine the effects of naturalness. The researchers found that perceived naturalness significantly affected perceived vitality but that it had no effect on mood or perceived restorativeness. Measuring brain activity through quantitative electroencephalography (qEEG), Johansson and colleagues [12] found that an intermediate level of forest biodiversity was appraised most favorably by participants, followed by high biodiversity, and lastly by low biodiversity. 

Clearly, the question of whether biodiversity facilitates psychological benefits has not been definitively answered. In examining the existing literature on this topic, Lovell et al. [16] identified several limitations, including the limited number of studies examining the issue; the heterogeneity of research designs and measures of well-being and the environment present in that small body of work; and the inherent complexity in the relationship between biodiversity and human health. In their review, the researchers conclude that there is a lack of evidence as to whether biodiversity is causally related to human well-being. Randomized trials are of great value in exploring causality, particularly in a field where the independent variable cannot be experienced in situ in the absence of countless extraneous variables, as is the case with biodiversity. To that end, we conducted a randomized Immersive Virtual Environment (IVE) experiment in which we first induced stress in participants using an IVE Trier Social Stress Test and examined recovery in three IVE natural environments depicting ordinal levels of biodiversity as well as one IVE urban environment. In order to increase immersion in the IVE environments, auditory representations of species richness and increasingly diverse olfactory stimuli were also provided.

Specifically, we sought to address the following research questions:

1. Is stress induced by a novel IVE Trier Social Stress Test (TSST-IVE)?
2. Does recovery from induced stress differ between IVE “urban” and “natural” environments?
3. Is stress recovery influenced by biodiversity?
4. Does perceived biodiversity accurately reflect actual biodiversity?
5. Does multisensory stimulation affect perceptions of biodiversity?
6. Does multisensory stimulation influence recovery from induced stress?

## 2. Materials and Methods

### 2.1. Participant Recruitment

Participants were initially invited to take part in the experiment via a department-wide email at the principal researcher’s institution. In total, 52 participants were included in the study. The majority of participants were university staff, but the sample also included undergraduate and postgraduate students as well as members of the public who had heard of the experiment via word-of-mouth. Twenty-nine percent of participants had a professional and/or tertiary education background in engineering; 21% had a background in science; 18% in business or administration; 12% in education; and the remaining participants had a range of professional backgrounds including trades, information technology, psychology, and journalism. Potential participants completed an online screening questionnaire to exclude individuals with medical issues such as hypertension, epilepsy, and cardiac health problems. The gender distribution was approximately even (53.8% female, 46.2% male), with a mean age for all participants of 37.6 (SD = 10.6). The screening questionnaire also collected data about participants’ current levels of perceived stress using the 10-item Perceived Stress Scale [22]; their emotional connectedness to nature using the 6-item short form of the Nature Relatedness Scale [23]; their frequency of nature visits, and an indication of their level of knowledge about ecology and biodiversity. The latter was achieved through two questions: (1) from a list of 13 ecology-related words (e.g., “ecosystem service” and “habitat fragmentation”), participants were asked to indicate which words they would feel confident explaining the meaning of to another person and, (2) from a list of six “consequences of biodiversity loss” containing three incorrect answers, participants were asked to select which ones they believed were likely to represent future consequences of biodiversity loss.

### 2.2. IVE Environments

The Immersive Virtual Environments were 360 × 270-degree videos of real parks located in metropolitan Adelaide, South Australia. All IVEs were filmed on days with similar weather conditions (partly cloudy) and with a slight breeze (to ensure all videos exhibited some movement). They were also filmed at the same time of year (late winter/early spring) to ensure the vegetation, particularly grasses, had not browned off due to dry weather. To ensure the experiment would test the effects of biodiversity (while using real environments and non-manipulated imagery), every effort was put into controlling for extraneous variables in the IVEs. Selection of an appropriate filming location within each environment was based on the following:

- On-site assessment of structural heterogeneity using a rapid assessment tool (described in Reference [24].
- The absence of people within the scene.
- The exclusion of elements that were visually inconsistent with the level of biodiversity desired (e.g., although most urban settings contain some vegetation, the selected urban scene did not contain vegetation to represent a base level of zero species diversity).
- Similar spatial configurations (i.e., open yet defined environments with “relatively smooth ground texture and trees that help define the depth of the scene” [25] (p. 48)). Although the ground is less smooth in the high biodiversity scene, it is still traversable. The role of “trees” as a spatial element in the urban environment is filled by a tower and tall columns.
- Similar styles of natural environment, i.e., predominantly native plant species, and not overly manicured (e.g., all exhibit dead/bare patches in ground cover and do not contain formal garden beds or plantings).
- The exclusion of animal species in all videos.

**Figure 1:**
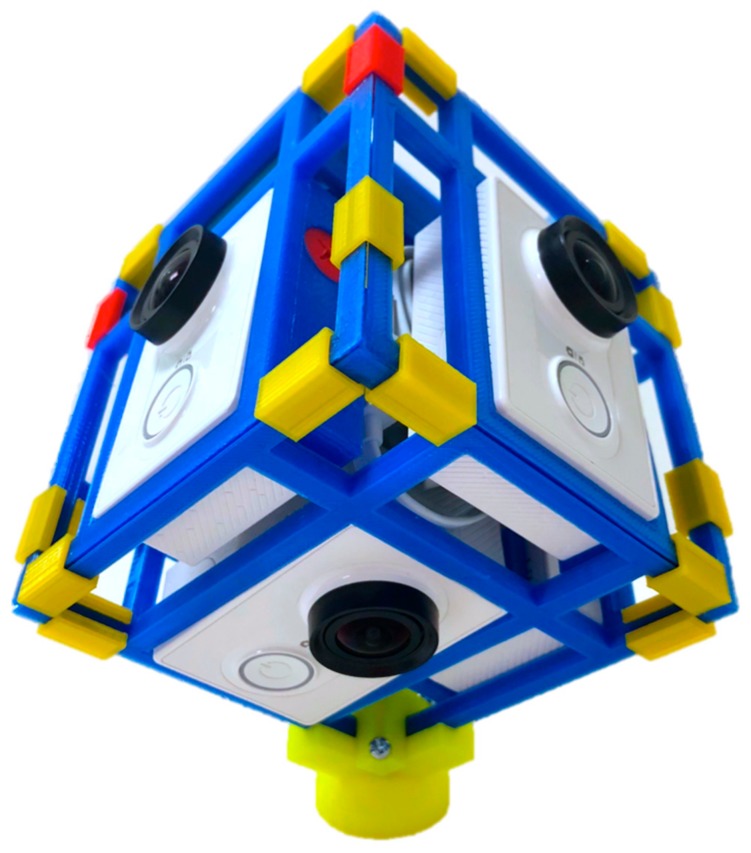
The 360-degree camera mount and Xiaomi Yi cameras used to film the Immersive Virtual Environments (IVEs).

The IVEs were filmed using six Xiaomi Yi high definition action cameras (similar to a GoPro), mounted on a 3D printed mount (Figure 1) and a monopod. The six individual videos were stitched into a single 360-degree video using Autopano Video Pro software. Participants viewed the IVEs using an Oculus Rift head-mounted display (HMD).

**Figure 2:**
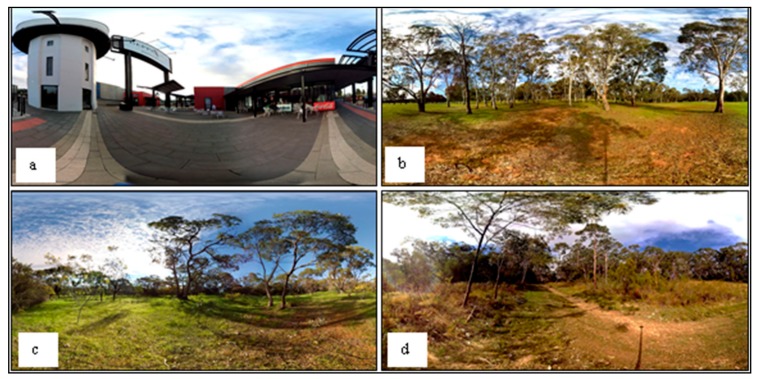
Example image of the four IVEs used in the experiment: (**a**) Urban; (**b**) low biodiversity; (**c**) moderate biodiversity; (**d**) high biodiversity. Note: as the IVEs are spherical worlds, two-dimensional rectangular images appear distorted.

There were five Immersive Virtual Environments (IVEs) included in the study. These are described below, and a summary of stimuli used in each is provided in Table 1:

**Urban**: a multisensory urban IVE (Figure 2a). Care was taken to select a scene that was visually interesting, was clean, and exhibited structural variation, as with the nature scenes. It comprised an outdoor shopping mall with *al fresco* dining areas, a tower, and tall signs.

**Low**: a multisensory low biodiversity IVE (Figure 2b). This scene consisted of two vegetation layers, i.e., two species of *Eucalyptus* trees taller than 10 m high, and ground layer vegetation less than 10 cm high. The total number of structural elements (including non-vegetation elements) was four. 

**Moderate**: a multisensory moderate biodiversity IVE (Figure 2c). This scene consisted of four vegetation layers, i.e., two species of Eucalyptus trees taller than 10 m high, a species of *Acacia* shrub less than 5 m tall, small saplings, and ground layer vegetation less than 10 cm high. The total number of structural elements was eight.

**High-M**: a multisensory high biodiversity IVE (Figure 2d). This scene consisted of seven vegetation layers, i.e., two species of Eucalyptus trees taller than 10 m high, a species of *Acacia* tree less than 10 m tall, shrubs taller than 5 m high, shrubs less than 5 m high, small saplings, ground layer vegetation taller than 10 cm, and ground layer vegetation less than 10 cm high. The total number of structural elements was 15.

**High-V**: a visual-only version of the high biodiversity IVE, without auditory or olfactory stimulation (Figure 2d).

### 2.3. Auditory Stimuli

The IVEs were paired with soundscapes that reflected ordinal levels of biodiversity. Soundscapes were recorded using a Zoom H6 recorder mounted on a tripod, with dual microphones (one forward-facing, unidirectional microphone and one side-facing, bidirectional microphone). The soundscapes were not recorded in the same locations as the IVE videos, with the exception of the urban scene. In order to control for sounds that were not consistent with the environment depicted (e.g., traffic, machinery, and people), a base layer soundscape (low biodiversity) was recorded in a rural setting. It contained only “natural” sounds such as birds and the sound of breeze rustling through tree leaves. The soundscape contained a single native bird species, the Australian magpie (*Gymnorhina tibicen*), and this soundscape became the base recording upon which the medium and high biodiversity soundscapes were built. As the ordinal level of biodiversity increased, the base recording was mixed with additional recordings of other native bird species, such as eastern rosellas (*Platycercus eximius*) and kookaburras (*Dacelo novaeguineae*), so that the high biodiversity soundscape contained the greatest number of bird species. Mixing/layering of recordings was done using Audacity software.

### 2.4. Olfactory Stimuli

Due to budget constraints, olfactory stimulation was incorporated through a selection of scents from the Demeter Fragrance Library rather than through a dedicated scent delivery system. A range of 16 “nature”-based scents (e.g., several types of grass, soils, leaves, and flowers) and four “urban”-based scents (e.g., dust and turpentine) were initially tested on a convenience sample of 11 people who were asked to describe each fragrance and the type of environment they believed it was consistent with. Participants were not prompted with scent types or settings, but variations of natural settings (e.g., park, forest, and nature) and urban settings (e.g., city, road, and machine) were accepted as “natural” or “urban”, respectively. Seven scents were correctly identified in type (e.g., grass) and setting by all respondents and were used in the IVE experiment. Similar to the biodiversity soundscapes, as the desired biodiversity level increased, so too did the number of scents, which were applied to cotton pads and attached to the head-mounted display during the final phase of the experiment. The urban and low biodiversity scenes contained a single scent, and the high biodiversity scene contained three different natural scents.

### 2.5. IVE Trier Social Stress Test

**Figure 3:**
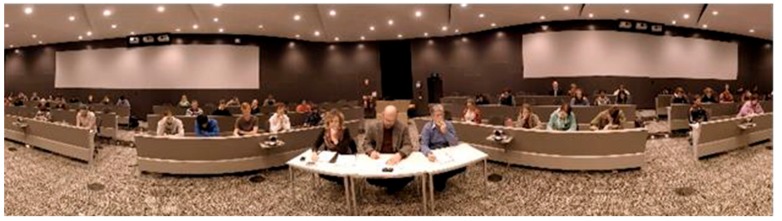
The IVE Trier Social Stress Test (TSST-IVE) used in the study, depicting a real 360-degree audience and a panel of three judges.

The Trier Social Stress Test (TSST) [26] is one of the most widely used protocols for inducing psychological stress in laboratory settings. It consists of several stages, two of which involve the participant verbally presenting in front of an audience. Traditionally, the audience in the TSST is a panel of three trained actors who maintain a neutral expression throughout the procedure. Although the TSST is effective at inducing a stress response, it has several limitations: (1) the cost/logistical difficulty associated with requiring separate rooms for the participant and the audience prior to the speech stages, (2) the human effort involved in having multiple actors present for each participant, (3) the time needed to train the actors to maintain neutral behaviors throughout the TSST, and (4) the possibility that the actors will not behave in the same way for each participant [27]. To overcome these limitations, researchers have begun using virtual reality versions of the TSST (TSST-VR) instead of using a live audience, e.g., References [28,29,30]. Most previous versions of the TSST-VR have used a computer-generated virtual audience, which may appear quite artificial to participants. In the present study, we used an IVE version of the panel, as shown in Figure 3. The TSST-IVE was a 360-degree video of an oval-shaped lecture hall containing a central stage surrounded by seating on all sides. A panel of three judges was seated in front of the participant, and a larger audience surrounded the participant in 360 degrees (i.e., participants were aware that the audience was also watching from behind them, and they were able to turn in any direction to view those members of the audience).

### 2.6. Measures

#### 2.6.1. Physiological Data

Electrodermal activity (EDA) and heart rate (HR) were recorded using Empatica E4 wristbands. An earlier version of the wristband is described in Reference [31]. An E4 wristband was applied to each wrist, as EDA signals may be weaker one side of a person’s body, e.g., References [32]. Although EDA is traditionally measured at the fingers or palms, wrist EDA measurements have been shown to correlate with finger EDA measurements during emotional responses to videos [33]. Heart rate was recorded in beats per minute (BPM) at a rate of 1Hz, and EDA was recorded in microSiemens (μS) at a rate of 4Hz.

#### 2.6.2. Psychological Data

Psychological data were measured using separate Visual Analogue Scales (VAS) for perceived stress, anxiety, insecurity, calmness, and happiness. The VAS were 100-mm long horizontal lines, with a label at each end, e.g., “not at all anxious” to “extremely anxious”. Participants completed the five VAS at the end of each stage of the TSST (i.e., they completed the VAS five times during the experiment). Although multiple-item scales are preferable, studies have shown that the use of single-item response scales can accurately measure well-being, e.g., stress [34], anxiety [35], and happiness [36].

#### 2.6.3. Perceptions

Perceived “plant and animal diversity” was measured using a five-point scale as well as the four-item Biodiversity Experience Index (BEI) [37]. Perceived “nativeness” of the species in the environment, perceived “beauty”, and perceived “weed presence” were also measured on five-point scales. 

Participants’ sense of presence in the IVEs was measured using a modified version of Kim and Biocca’s [38] telepresence questionnaire. Presence is defined as “the subjective experience of being in one place or environment, even when one is physically situated in another” [39] (p. 225). We modified Kim and Biocca’s [38] telepresence questionnaire by replacing references to “broadcast” or “television” with “virtual environment”. We selected this questionnaire as it focused on participants’ sense of feeling like they were in the IVE and did not include questions related to having control over events in the environment such as in Witmer and Singer [39].

#### 2.6.4. Experiment Procedure

Each participant took part in the experiment individually. The experiment closely followed the original TSST procedure [26] and had five stages (for graphical depiction, see Appendix A): 

##### Relaxation

Upon arriving at the Virtual Reality (VR) laboratory, each participant was greeted by two researchers who were wearing white lab coats, as per Kirschbaum et al. (1993). Participants were seated in a waiting area that was partitioned off from the rest of the VR laboratory. Their wrists were cleaned with alcohol, and an Empatica E4 wristband was applied to each wrist. A 15-minute baseline was recorded, after which they completed the first set of VAS (T1). Participants then joined the researchers in the VR lab, where they were asked to sit at a desk that held a computer and an Oculus Rift VR headset, prior to commencing the first part of the experimental task phase of the TSST. 

##### Anticipation

A researcher explained to participants that they had five minutes to mentally prepare a five-minute speech about their positive and negative personality traits. The participant was told that the speech would be filmed and reviewed by university students who were taking part in a public speaking workshop that semester. A video camera was placed on a tripod next to the desk in view of the participant. The second set of VAS were completed after five minutes of preparation time (T2).

##### Speech

Participants were told that they should speak about their positive and negative personality traits for the entire five-minute period. The VR headset was put on the participant, and the TSST-IVE began. If participants stopped talking for more than 10 seconds, they were reminded that they should continue speaking for the entire five-minute period. Upon completing their presentation, the VR headset was removed and the third set of VAS were filled in (T3).

##### Arithmetic

Participants put the VR headset on again and were asked to count backwards from 1022 in counts of 13. They were informed that, if they made a mistake, they would be asked to begin counting from 1022 again. After five minutes, the headset was removed so that the fourth set of VAS could be completed (T4).

##### Recovery

In the final phase of the experiment, participants once again donned the VR headset and spent five minutes in one of the IVEs outlined previously. After the recovery phase, participants were asked to complete the fifth set of VAS (T5), followed by a questionnaire regarding their perceptions of the environment they had experienced.

#### 2.6.5. Data Analysis

Data were analyzed using SPSS statistics software version 24. Data screening was conducted to check the suitability of the data for further analysis. At each time point during the experiment, some measures were not normally distributed for at least one IVE group. Therefore, analyses under each objective are comprised of both parametric and nonparametric tests. Inspection of the EDA data revealed that EDA recordings for many participants were incomplete, and EDA data were therefore not included in the analysis. As heart rate was recorded continuously throughout the experiment, analyses were conducted using average heart rate during the final 60 seconds of each experimental phase.

Prior to conducting analyses related to each research objective, we tested whether there were significant differences in each well-being measure between the experiment groups at baseline (T1) to support further analysis. In all cases, normality of data was tested using the Shapiro–Wilk test. Baseline comparability between the different biodiversity groups was then investigated using either the one-way ANOVA procedure or the nonparametric Kruskall–Wallis test, as appropriate. Where significant results were obtained from the Kruskall–Wallis test, post hoc analyses were performed using Dunn’s [40] procedure with a Bonferroni correction applied to adjust for multiple comparisons.

At T1, only perceived happiness was normally distributed in all five groups. A one-way ANOVA was conducted to determine if baseline levels of perceived happiness differed between the five IVE groups. There was homogeneity of variances, as assessed by Levene’s test of homogeneity of variances (*p* = 0.941). There were no significant differences in perceived happiness at T1 between the groups (F4,47 = 0.10, *p* = 0.982). 

Perceived stress, anxiety, insecurity, calmness, and HR at baseline were not normally distributed in at least one IVE group. A Kruskal–Wallis H test was therefore run to determine if there were significant group differences in the five measures at baseline, with the result shown in Table 2. There were no significant differences in median scores between groups at T1, suggesting the different biodiversity groups had sufficiently comparable scores at the baseline to support a comparative analysis.

Initial checks also showed that were no significant differences in nature relatedness or background stress levels between groups, as determined by one-way ANOVA for the Nature Relatedness Scale (F4,47 = 0.497, *p* = 0.738) and for the Perceived Stress Scale (F4,47 = 0.756, *p* = 0.559). 

A brief overview of the statistical tests used in this study is shown under the research questions they addressed:

**Research question 1:** Because the TSST-IVE, which we used to induce a stress response, generates multiple dependent observations, differences in self-reported stress, anxiety, insecurity, calmness, happiness, and HR were calculated subtracting baseline (T1) from the speech phase (T3) of the experiment for each participant. The statistical difference between T1 and T3 was tested using paired-samples t-tests for those measures that met the test’s assumptions, namely heart rate, self-reported stress, and happiness. Self-reported anxiety, calmness, and insecurity were analyzed using Wilcoxon signed-rank tests.

**Research question 2:** In examining stress recovery in “urban” and “natural” IVEs, separate independent-samples t-tests were used for well-being measures that met the test’s assumptions of normality, no outliers, and homogeneity of variance, namely perceived stress, anxiety, and calmness. Where the assumptions of the independent samples t-test were not met, we conducted Mann–Whitney U tests.

**Research question 3:** To examine the effect of biodiversity on well-being recovery, a one-way ANCOVA (Analysis of Covariance) was used for those measures that met the test’s assumptions of normality, no outliers, and homogeneity of variance, namely self-reported stress, anxiety, and calmness. The remaining well-being measures were assessed using Kruskal–Wallis H tests.

**Research question 4:** Perceptions of biodiversity were not normally distributed; thus, to compare perceived biodiversity between the four multisensory IVE groups (representing ordinal levels of biodiversity), we used Kruskal–Wallis H tests.

**Research questions 5 and 6:** A Mann–Whitney U test was used to compare perceptions of biodiversity between the visual-only high biodiversity IVE and the multisensory high biodiversity IVE. Mean differences in heart rate (HR) between T4 and T5 for these two groups were normally distributed; thus, independent samples t-tests were used. The self-reported well-being measures were assessed using Mann–Whitney U tests. Direct comparisons of “presence”—the feeling of being in the virtual world and not in the real world—was also examined in relation to the influence of multisensory stimulation (research questions 5 and 6). As the data were normally distributed and did not contain outliers, differences in presence were compared using an independent samples t-test.

## 3. Results

### 3.1. Psychological and Physiological Responses to the TSST-IVE

**Figure 4:**
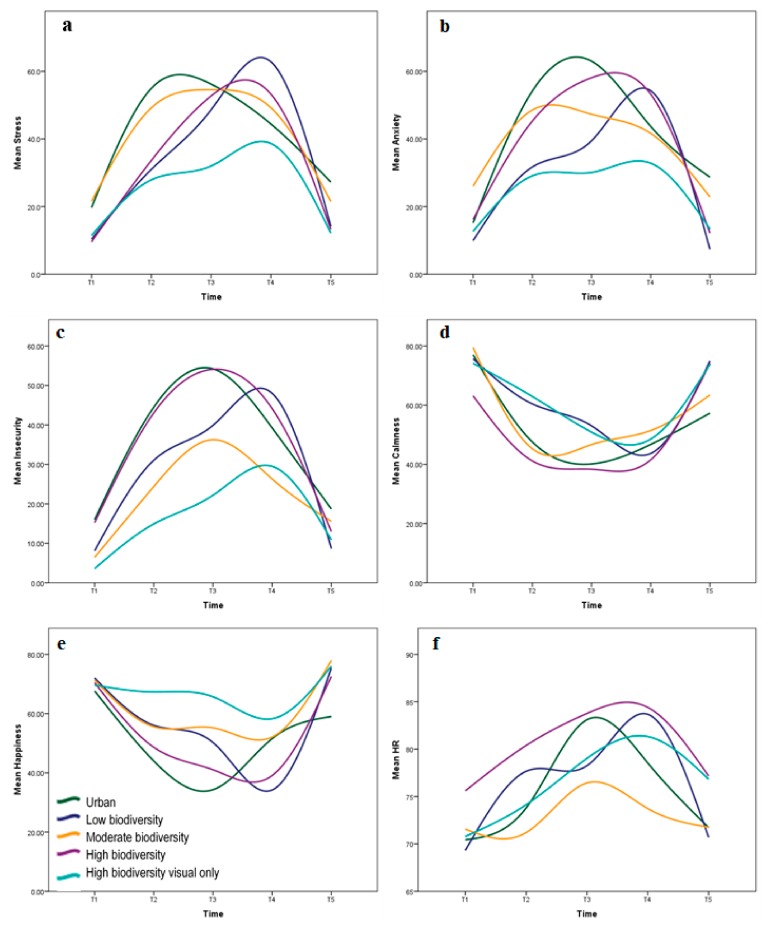
Aggregated well-being measures over the course of the IVE experiment for (**a**) self-reported stress; (**b**) self-reported anxiety; (**c**) self-reported insecurity; (**d**) self-reported calmness; (**e**) self-reported happiness; and (**f**) heart rate.

The differences in well-being measures between baseline (T1) and the speech phase (T3) for the TSST-IVE were calculated for each participant. Aggregated results are presented as Figure 4.

There were no outliers in the differences between scores for stress, happiness, and HR, as assessed by visual inspection of boxplots. The difference scores for each measure at T1 and T3 were normally distributed, as assessed by Shapiro–Wilk’s test (*p* = 0.177 for stress, *p* = 0.279 for happiness, and *p* = 0.758 for HR). Paired-samples t-tests showed that performing a five-minute speech in front of the IVE audience elicited a significant mean increase in perceived stress and HR and a significant decrease in perceived happiness, as shown in Table 3. There was a large effect size (Cohen’s d).

The differences between T1 and T3 for perceived anxiety, insecurity, and calmness were not normally distributed, as assessed by Shapiro–Wilk’s test (*p* < 0.05). Wilcoxon signed-rank tests showed that performing a five-minute speech in front of the IVE audience elicited a significant increase in perceived anxiety and insecurity and a significant decrease in perceived calmness amongst participants (Table 4). Thus, all measures of well-being changed significantly after commencement of the TSST-IVE.

### 3.2. Stress Recovery in “Urban” and “Natural” IVEs

To determine whether stress recovery differed significantly between the “urban” IVE and each of the multisensory “nature” IVEs, recovery scores were calculated from the difference in well-being responses between T4 and T5.

There were significant differences in recovery between the urban and low biodiversity IVEs for perceived stress, anxiety, and happiness (Table 5 and Table 6). There were no significant differences between the urban and moderate biodiversity IVEs or between the urban and high biodiversity multisensory IVEs. For perceived insecurity, calmness, and HR, there were no significant differences in recovery between the urban and nature IVEs. Although most differences did not reach significance, out of 18 paired comparisons between “urban” and “nature” IVEs, the urban group experienced the least effective recovery in 15 comparisons.

### 3.3. Does Stress Recovery Differ Amongst IVEs Representing Ordinal Levels of Biodiversity?

**Figure 5:**
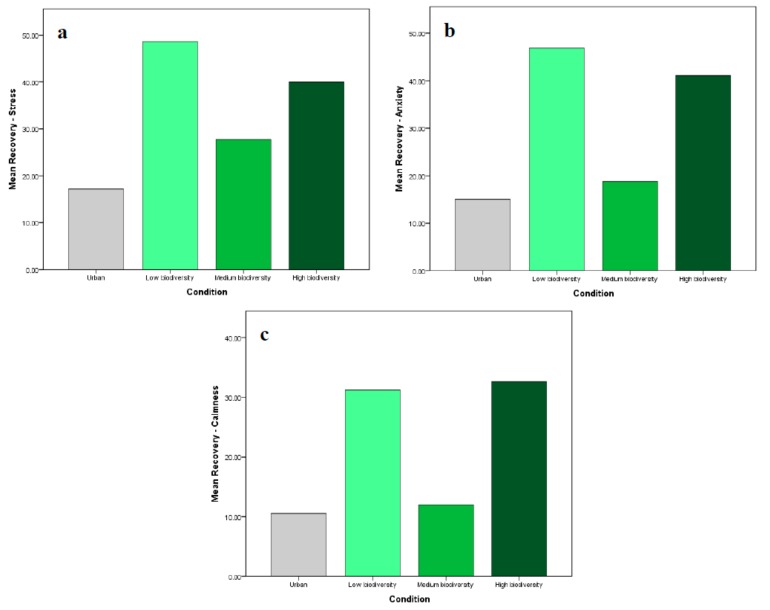
Mean recovery scores (from T4 to T5) for (**a**) perceived stress; (**b**) anxiety; and (**c**) calmness in the multisensory IVEs.

We sought to determine whether there was a statistically significant difference in stress recovery between the three multisensory biodiversity IVEs. There were no significant differences in recovery scores for perceived stress, anxiety, or calmness between the low, moderate, and high biodiversity environments (*p* > 0.05). However, pairwise comparisons using t-tests showed that perceived anxiety recovery scores were significantly greater in the low biodiversity environment than in the moderate biodiversity environment (*p* = 0.038) (Figure 5). 

**Figure 6:**
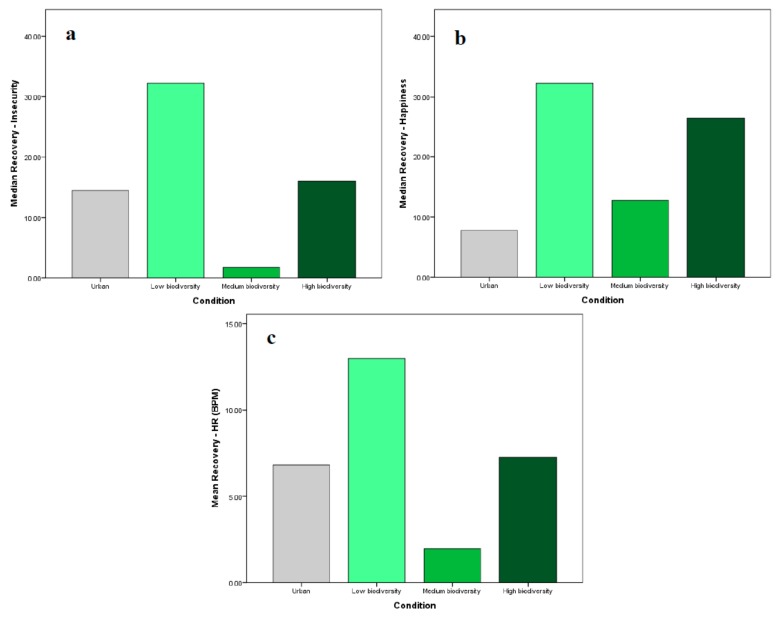
Median recovery scores (from T4 to T5) for (**a**) perceived insecurity; (**b**) happiness; and (**c**) HR in the multisensory IVEs.

Kruskal–Wallis H tests showed no significant differences in perceived insecurity and happiness recovery scores between the biodiversity IVEs; however, there were significant differences in median HR recovery scores (χ2(2) = 9.234, *p* = 0.007) as shown in Figure 6. A post hoc analysis revealed that median HR recovery was significantly greater in the low biodiversity group (20.50 BPM reduction) than in the moderate biodiversity group (10.20 BPM reduction). Thus, there was a significant difference in some measures of stress recovery (i.e., perceived anxiety and HR) between the low biodiversity and moderate biodiversity scenes, with the low biodiversity scene eliciting greater recovery from induced stress in both cases. Of the multisensory biodiversity IVEs, the moderate biodiversity environment consistently elicited the least effective recovery. 

A one-way ANCOVA was used to examine the effect of biodiversity on recovery scores when controlling for several participant attributes, including nature relatedness (measured using the Nature Relatedness Scale), knowledge of ecological concepts, knowledge of consequences of biodiversity loss, and general perceptions of day-to-day stress (measured using the Perceived Stress Scale (PSS) in the screening questionnaire). There were no significant differences when controlling for the tested covariates either individually or collectively.

### 3.4. Does Multisensory Stimulation Influence Recovery from Induced Stress?

**Figure 7:**
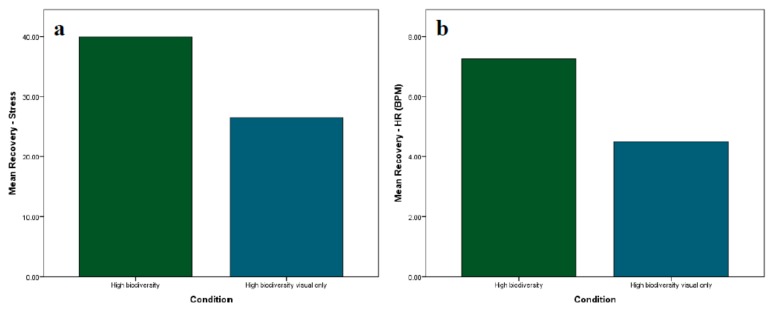
Mean recovery for (**a**) self-reported stress and (**b**) heart rate in the multisensory high biodiversity IVE and the visual-only high biodiversity IVE.

Differences in recovery from T4 to T5 between the multisensory high biodiversity IVE and the visual-only high biodiversity IVE were examined. Perceived stress and HR were normally distributed in both groups and did not contain outliers. Independent-samples t-tests showed that, although perceived stress and HR recovery were greater in the multisensory IVE (mean decrease in perceived stress of 39.95 and mean decrease in HR of 7.25 BPM) than the visual-only IVE (mean decrease in perceived stress of 26.45 and mean decrease in HR of 4.49 BPM), as shown in Figure 7, the differences were not statistically significant (*p* > 0.05).

**Figure 8:**
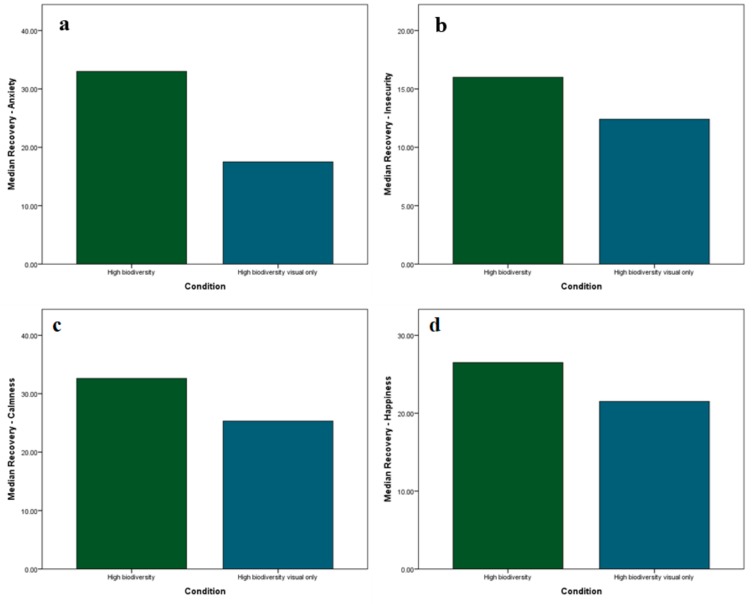
Mean recovery for self-reported (**a**) anxiety; (**b**) insecurity; (**c**) calmness; and (**d**) happiness.

Mann–Whitney U tests were used to examine whether multisensory stimulation affected recovery scores for self-reported anxiety, insecurity, calmness, and happiness. Median anxiety recovery scores were significantly greater in the multisensory high biodiversity IVE (33.00) than the visual-only IVE (17.50), with *U* = 90.50, *z* = 1.971, *p* = 0.047. Recovery scores were consistently greater in the multisensory IVE than the visual-only IVE (Figure 8); however, the difference in recovery was not statistically significant for perceived insecurity, calmness, or happiness (*p* > 0.05).

### 3.5. Influences of Environment on Perceptions of Nature

We explored whether perceptions of biodiversity, nativeness, beauty, and weed presence differed between the different levels of biodiversity. Perceptions were reported using 5-point scales. Biodiversity perceptions were also ascertained using the 4-item Biodiversity Experience Index (BEI).

**Figure 9:**
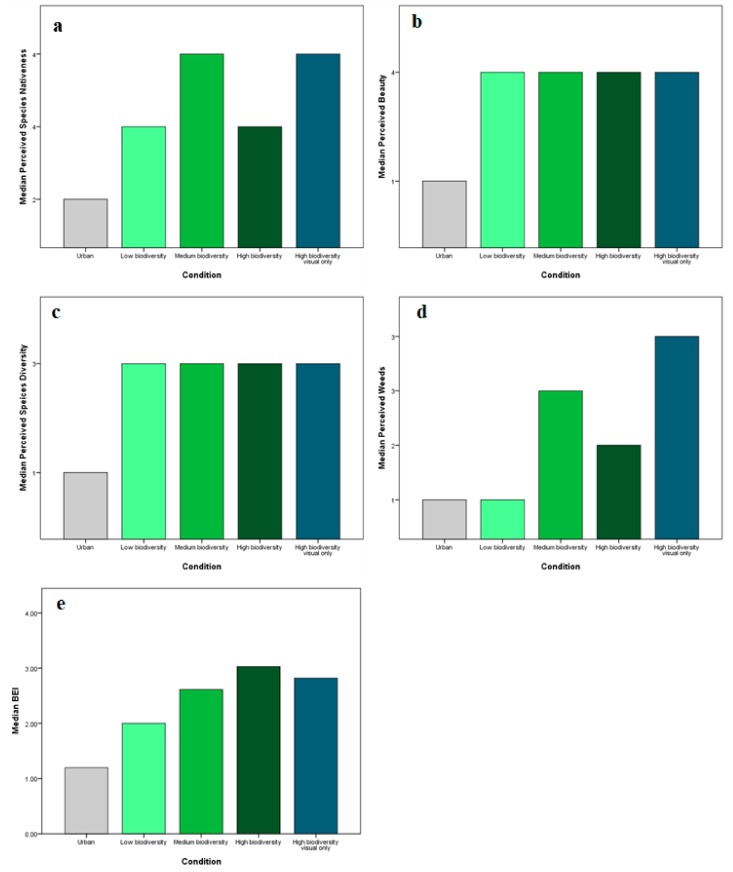
Median perceptions of (**a**) nativeness; (**b**) beauty; (**c**) species diversity; (**d**) weed presence; and (**e**) Biodiversity Experience Index (BEI) in the virtual environments.

Due to the nonparametric nature of the data, Kruskal–Wallis tests were employed to test for differences in perceptions of biodiversity, nativeness, beauty, and weed presence between the IVEs. All five biodiversity groups were included. As expected, the urban environment was perceived as significantly less biodiverse, less native, and less beautiful than all of the natural environments (*p* < 0.01). Only the BEI scores were found to differ significantly between the ordinal levels of multisensory biodiversity (Figure 9e). Median BEI scores were significantly different between the three levels of biodiversity (χ2(2) = 8.576, *p* = 0.014). A post hoc analysis revealed a significant difference in median BEI scores only between low (2.0) and high biodiversity (3.03).

**Figure 10:**
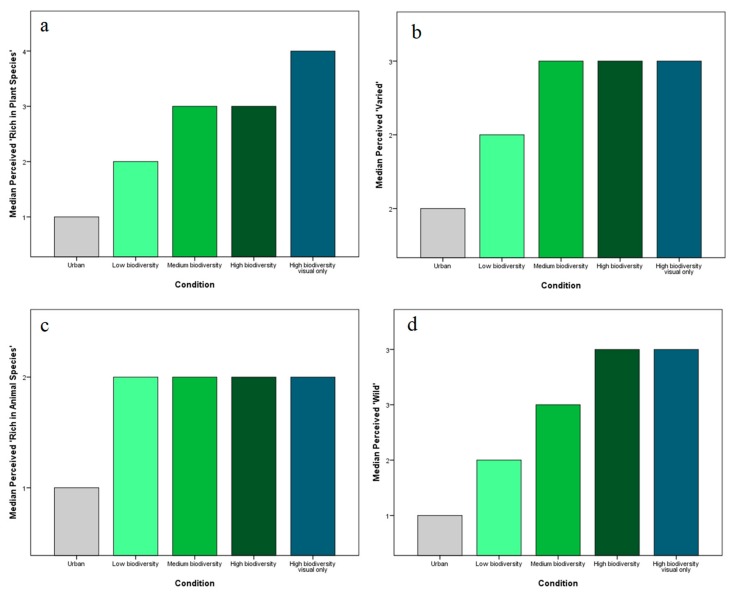
Median scores for the individual components of the Biodiversity Experience Index (BEI); (**a**) Median Perceived ’Rich in Plant Species’; (**b**) Median Perceived ’Varied’; (**c**) Median Perceived ’Rich in Animal Species’; (**d**) Median Perceived ’Wild’.

Exploring responses to the individual components of the BEI provides further insight into how the IVEs were perceived (Figure 10). Interestingly, median perceived animal richness does not differ significantly between the three levels of biodiversity. Animal richness is equally high in the visual-only high biodiversity IVE, in which no animals were depicted (i.e., because the audio recordings provided the only representation of wildlife in the IVEs). Conversely, Figure 10a shows perceived plant richness is greatest in the visual-only high biodiversity IVE, suggesting participants may have paid greater attention to the visual attributes of the IVE without the distraction of auditory and olfactory stimuli. The stepwise increase shown in Figure 10d suggests that perceived “wildness” best reflects the ordinal levels of biodiversity included in the experiment.

### 3.6. Influences of Multisensory Stimulation on Perceptions of Nature

We explored whether perceptions of biodiversity, nativeness, and beauty differed between the multisensory high biodiversity environment and the visual-only high biodiversity environment. As the data were not always normally distributed in both groups, we used the Mann–Whitney U test to determine whether there were significant differences in perceptions of the two environments but found no significant differences (*p* > 0.05). 

### 3.7. Influences of Multisensory Stimulation on Presence in Virtual Environments

We sought to determine whether participants’ sense of immersion in the IVE was greater in the multisensory high biodiversity environment than in the visual-only environment due to the lack of auditory and olfactory stimulation in the latter group. Presence data were normally distributed for both groups, as determined by Shapiro–Wilk’s test (*p* = 0.454 for multisensory and *p* = 0.087 for visual-only). A t-test was applied to determine whether mean presence scores differed between the multisensory and visual-only high biodiversity IVEs. The results were not significant (t(20) = 0.095, *p* = 0.925), and in fact, mean presence scores were almost identical for the two groups, with a difference of only 0.023 (95% CI, −0.477, 0.522).

## 4. Discussion

We conducted an exploratory Immersive Virtual Environment (IVE) experiment to test the effects of biodiversity on recovery from induced stress in 52 participants. Furthermore, we examined whether providing a multisensory nature experience affected recovery from induced stress and/or influenced perceptions of natural environments in order to guide future experimental studies. To our knowledge, it is the first stress intervention trial in this area, and whilst the sample size was small, the results that did reach statistical significance had a large effect size. Some of the finer details of our exploration did not reach statistical significance but may arguably have done so with a larger sample size. We believe that the consistency in the direction of well-being responses across multiple measures, both self-reported and physiological, provides useful insight into the effects of biodiversity on human well-being.

### 4.1. Stress Inducement via an IVE Trier Social Stress Test

The TSST-IVE was effective at significantly increasing heart rate and self-reported stress, anxiety, and insecurity and at significantly decreasing self-reported calmness and happiness. Using the traditional TSST, which is considered by many to be the gold standard in laboratory-based stress inducement, e.g., Reference [41], was not a financially or logistically viable option for this study. Because researchers are needed to conduct the experiment and additional staff or trained volunteers are required to play the role of audience/judges, the traditional TSST can be a costly and time-consuming protocol that may be out of reach for small research teams. As such, the TSST-IVE could be a feasible method for future experimental research in nature and well-being studies and can be produced by researchers for their own studies without in-depth knowledge of digital design or computer programing. As described by Smith [42], IVE technologies are becoming increasingly cost-effective and simple to use. Examining causal relationships between human well-being and nature attributes such as biodiversity will require experimental research; thus, any method that improves the efficiency or ease with which such studies can be conducted is of great benefit to this body of work.

### 4.2. Stress Recovery in “Urban” and “Nature” IVEs

Recovery after the TSST was least effective in the urban IVE for all measures of well-being, with the exception of heart rate and self-reported insecurity. The differences in recovery between urban and natural scenes were only significant for self-reported stress, anxiety, and happiness, which showed the greatest improvement in the low biodiversity IVE. There were no significant differences in well-being between the urban and moderate or high biodiversity IVEs; however, we consider (a) that the savanna-like landscape of the low biodiversity IVE is most reflective of the natural environments used in much past research comparing well-being in urban and natural environments [3] and (b) that, despite few significant differences, the urban environment was generally the least restorative environment. In that regard, the findings are consistent with the long-established premise that natural environments are more restorative than built environments and therefore support the use of IVE as a valid and effective approach to measure restoration associated with natural environments.

### 4.3. Biodiversity Perceptions and Well-Being Responses to IVEs Differing in Biodiversity

In all but one measure (calmness), the low biodiversity scene elicited the greatest improvement in well-being during the recovery stage. However, the finding does not appear to be related to biodiversity per se, as the moderate biodiversity IVE was invariably the least restorative of the natural environments. There may be thresholds of species richness or structural heterogeneity, above which an environment becomes less restorative, but in that case, one would expect the high biodiversity environment to be perceived as equally restorative as the moderate biodiversity environment or less so, which was not the case. Furthermore, relative to the other two nature scenes, the biodiversity level (BEI) for the moderate biodiversity IVE was perceived accurately by participants, suggesting its species richness was not the reason it was responded to so poorly. Although some bird calls may be perceived negatively by people [43], we kept in mind that each bird sound and scent included in the moderate biodiversity experience was also present in the multisensory high biodiversity environment and that, thus, neither auditory nor olfactory stimuli were likely to explain participants’ responses. This might be expected given the strong priority placed on visual stimuli in humans (as opposed, for example, to smell in many animals). Examining the visual components of each of the scenes revealed no particular attribute that we might expect to cause the scene to be responded to as poorly as it was in comparison to the other nature scenes. The scene is a brighter green than the other IVEs and is the only scene to contain a dense shrub in the foreground. We examined perception responses to shed some light on why the moderate biodiversity IVE was the least restorative. Of the multisensory IVEs, the moderate biodiversity environment was perceived as having the most weeds and as being the most native. Perhaps it was the incongruence between those two perceptions that led to the scene’s lack of restorativeness. Whilst the tree and shrub species present in the scene are all native, the brightness of the ground cover (likely introduced grass species) may be perceived as being at odds with the native surroundings. Grose [44] suggests that the color of the natural environments with which we are familiar play a fundamental role in both our sense of place and in biological conservation. She suggests, “Colour can warn us about conservation because we can see it. The colours of our major plant species tell us something about our place, our landscape, our geographic region; the colours are likely to be distinct and different from other places, whether subtly or greatly” [44] (p. 161). Again, this is consistent with evolutionary theory, as humans may have evolved color vision to facilitate food finding, e.g., Reference [45]. Thus, our observation that the moderate biodiversity scene was a brighter green than the other nature scenes and participants’ perception that it contained more weeds may be linked. Given that our participants were highly educated individuals, many of whom work in environment-related disciplines, further research would be required to explore whether this seemingly subtle difference in the natural environments would affect other populations as strongly. 

### 4.4. Effects of Multisensory Stimulation on Biodiversity Perceptions and Well-Being Responses

Interestingly, the visual-only high biodiversity IVE was perceived to be more native and to also contain more weeds than the multisensory high biodiversity IVE, despite the visual stimuli being identical in both IVEs. In their VR experiment, Annerstedt et al. [46] found that a VR forest without auditory stimulation (nature sounds) may elicit feelings of uncertainty or unease that limit stress recovery. Similarly, in all measures of well-being, we found that participants in the multisensory environment experienced greater recovery than those in the visual-only environment. Furthermore, the difference in perceptions of nativeness and weed presence between the two environments suggests that a visual-only experience may elicit a greater level of scrutiny or visual assessment of the environment than that which occurs during a multisensory experience. Without the distraction of sounds and scents, participants may have focused more intently on the physical attributes of the environment, although they were not instructed to pay attention at all. It is of interest to note that perceptions of these ecological qualities differed when participants’ attention was focused on a single sense—perhaps being encouraged to take notice of nature and to put aside distractions can be beneficial from a conservation perspective. However, in terms of well-being, soft fascination is thought to be an essential component of restorative experiences [25] and the absence of additional sensory stimuli in the visual-only IVE may have resulted in participants not experiencing this effortless attention. Studies testing differences in recovery between participants instructed to take note of natural attributes and those who have received no instruction to do so would be of interest in determining whether we can be observant whilst deriving restorative benefits from nature. Ultimately, a population that benefits most from natural environments to which they do not pay close attention may fail to detect ecological change until it is too late. Perhaps our “environmental generational amnesia”, wherein each generation cannot see that their environment has degraded from the state it was in prior to their own existence [47], may begin to be stemmed by encouraging simple observation.

One of the strongest findings in this study was the clear appeal of a low biodiversity natural environment. Such an environment was perceived to be equally attractive as the more diverse environments and was the most restorative during recovery from induced stress. Possible reasons for people’s preferences for savanna-like landscapes such as the low biodiversity IVE are outlined by long-held evolutionary theories such as Appleton’s [48] prospect-refuge theory and Ruddell and Hammitt’s [49] habitat theory. In the present study, the participants in each group expressed a consistently high emotional connectedness to nature, so their responses were not related to their nature values. Furthermore, our participants were highly educated individuals, predominantly consisting of engineers and scientists, yet both their self-reported well-being and heart rate recovered best in a relatively depauperate environment, which, in reality, is a little-used area of incidental green space in an industrial area. Thus, their responses were not related to their knowledge of the importance of complexity or diversity in ecosystems. If we accept that people believe such environments are attractive and restorative and that they derive the most psychological benefit from them, the question then becomes what do we do about this? What do we do if human beings take the most pleasure from environments that provide little habitat to native species, that are vulnerable to the impact of stressors due to their lack of diversity, and that have limited capacity to continue providing ecosystem services to future generations? Would there be a difference if the test could be administered to toddlers (before imprinting with cityscapes as normal, healthy, happy places to be)? Furthermore, what do we do when environmental knowledge appears to have little influence on these perceptions and benefits? The participants in our study, for the most part, understood the importance of biodiversity and ecosystem health yet derived the greatest benefit from the least diverse and structurally complex environment. Education might not be key to improving perceptions of nature, but it might be key to public acceptance of environmentally conscious decision-making (e.g., Johansson, Gyllin, Witzell, and Küller [12] found that, despite differences in responses to forests with differing levels of biodiversity, acceptance of conservation measures did not differ between environments). Complexity and diversity are important attributes in many ecosystems, and our decision-making should reflect this, even if such environments are perceived less favorably by the public. The reversal of initiatives designed to increase biodiversity in urban parks has occurred in South Australia as a direct result of public opposition [50], and clearly, public opinion is a powerful force in decision-making, which cannot be discounted. The challenge lies in balancing those views against the consequences of not taking environmentally responsible action. In such cases where the two diverge, an ecologically literate public may be able to support sustainable management and planning decisions in spite of their personal preferences.

## 5. Conclusions

In this experiment, a stress response was successfully induced via an innovative Immersive Virtual Environment Trier Social Stress Test (TSST-IVE) and stress recovery took place during exposure to IVEs differing in plant and animal diversity. Biodiversity had a nonlinear effect on stress recovery, which was least effective in an urban environment, followed by a moderate biodiversity environment. Stress recovery was most effective in a low biodiversity environment. Multisensory stimulation (visual, auditory, and olfactory) led to improved stress recovery over a visual-only experience. In two identical visual scenes, the addition of visual and olfactory stimuli resulted in lower perceived plant species richness, lower perceived “nativeness”, and lower perceived “weed presence”. Multisensory stimulation had no effect on participants’ sense of immersion in the virtual environment. 

Returning to where we began this article, the unfortunate truth is as follows: that which is good for people is not always good for nature. While the landscape characteristics associated with greatest benefit attainment in this study (i.e., low biodiversity) were not reflective of conservation values, there is a significant risk to the environment if we do not value biodiversity or act in its interests. Ultimately, prioritising human well-being over ecosystem health will never be sustainable. To consider our findings from another perspective, we might suggest that, in reality, the preference exhibited towards low biodiversity environments may provide us an opportunity to better protect highly biodiverse environments. If people are more attracted to and derive greater benefit from environments exhibiting low complexity, then perhaps it is prudent in the short term to provide such landscapes as sacrificial entry points to begin building an appreciation and understanding of biodiversity. This needs to be done in conjunction with programs designed to increase diversity, wildness, and nativeness in urban areas, as it is only through such programs that we can hope to increase the public’s familiarity with these important concepts and can begin to unwind our generational amnesia and to curtail shifting baselines, so that when our environment changes, we notice. One who observes and who notices when ecosystems change with the seasons may start to notice when they change out of season. One who observes may learn, simply through experiencing nature in a more mindful way, to detect when familiar ecosystems are under pressure.

## Figures and Tables

**Table 1:** Breakdown of stimuli comprising each experimental condition.

Condition	Vegetation Layers	Natural Structural Elements	Bird Species in Soundscape	Scents
Urban	0	0	0	1
Low biodiversity	2	4	1	1
Moderate biodiversity	4	8	2	2
High biodiversity (M)	7	15	4	3
High biodiversity (V)	7	15	0	0

**Table 2:** Results of the Kruskal–Wallis H test showing no significant differences in well-being measures between biodiversity groups at baseline (T1).

Measure	*H*	*df*	*n*	*p*
Stress	1.189	4	52	0.135
Anxiety	4.987	4	52	0.298
Insecurity	1.619	4	52	0.805
Calmness	1.189	4	52	0.880
Heart rate (HR)	1.789	4	52	0.775

**Table 3:** Results of the paired t-tests showing significant differences in perceived stress, happiness, and HR as a result of the TSST-IVE.

Measure	T1 mean	T3mean	Mean Difference	95% CI	*t*	*df*	*p*	*d*
Stress	14.381	48.577	34.196	26.714, 41.678	9.175	51	<0.001	1.272
Happiness	70.212	49.452	20.760	13.703, 27.816	5.906	51	<0.001	0.820
HR	71.604	80.168	8.564	5.586, 11.541	5.775	51	<0.001	0.801

*Denotes significant difference (*p* < 0.05).

**Table 4:** Wilcoxon signed-rank tests showing significant differences in perceived anxiety, insecurity, and calmness as a result of the TSST-IVE.

Measure	T1 Median	T3 Median	Median Difference	*z*	*p*
Anxiety	10.00	48.50	32.75	5.751	< 0.001
Insecurity	2.75	36.00	24.50	5.730	< 0.001
Calmness	84.25	49.00	19.75	−5.371	< 0.001

*Denotes significant difference (*p* < 0.05).

**Table 5:** t-test results for the comparison of recovery scores for participants’ perceived stress, anxiety, and calmness between the urban IVE and each of the multisensory nature IVEs.

Measure	Urban Mean	Low Mean	Moderate Mean	High Mean	Mean Difference	95% CI	*t*	*df*	*p*	*d*
Stress	17.410	48.630	-	-	31.490	8.214, 54.766	2.842	18	0.011 *	
	17.410	-	27.690	-	10.550	−15.543, 36.646	0.849	18	0.407	
	17.410	-	-	39.945	22.806	−2.395, 48.006	1.894	19	0.074	
Anxiety	14.990	46.900	-	-	31.910	5.163, 58.658	2.506	18	0.022 *	
	14.990	-	18.800	-	3.81	−23.115, 30.735	0.297	18	0.770	
	14.990	-	-	41.064	26.074	−2.245, 54.393	1.927	19	0.069	
Calmness	10.550	31.240	-	-	20.690	−54.802, 13.422	1.274	18	0.219	
	10.550	-	12.000	-	1.450	−31.718, 28.818	0.101	18	0.921	
	10.550	-	-	32.618	22.068	−56.496, 12.360	0.196	19	0.189	

*Denotes significant difference (*p* < 0.05).

**Table 6:** Mann–Whitney U test results for the comparison of recovery scores for participants’ perceived insecurity, happiness, and HR recovery scores between the urban IVE and each of the multisensory nature IVEs.

Measure	Urban Median	Low Median	Moderate Median	High Median	*U*	*z*	*p*
Insecurity	14.500	32.250	-	-	65.50	1.172	0.247
	14.500	-	1.750	-	39.50	−0.794	0.436
	14.500	-	-	16.000	63.00	0.564	0.605
Happiness	7.750	32.250	-	-	19.00	−2.343	0.019 *
	7.750	-	12.750	-	30.50	−1.457	0.143
	7.750	-	-	26.500	29.50	−1.796	0.072
HR	6.818	10.795	-	-	58.00	0.605	0.579
	6.818	-	4.029	-	31.00	−1.436	0.165
	6.818	-	-	4.582	51.00	−0.282	0.809

*Denotes significant difference (*p* < 0.05).

**Figure A1:**
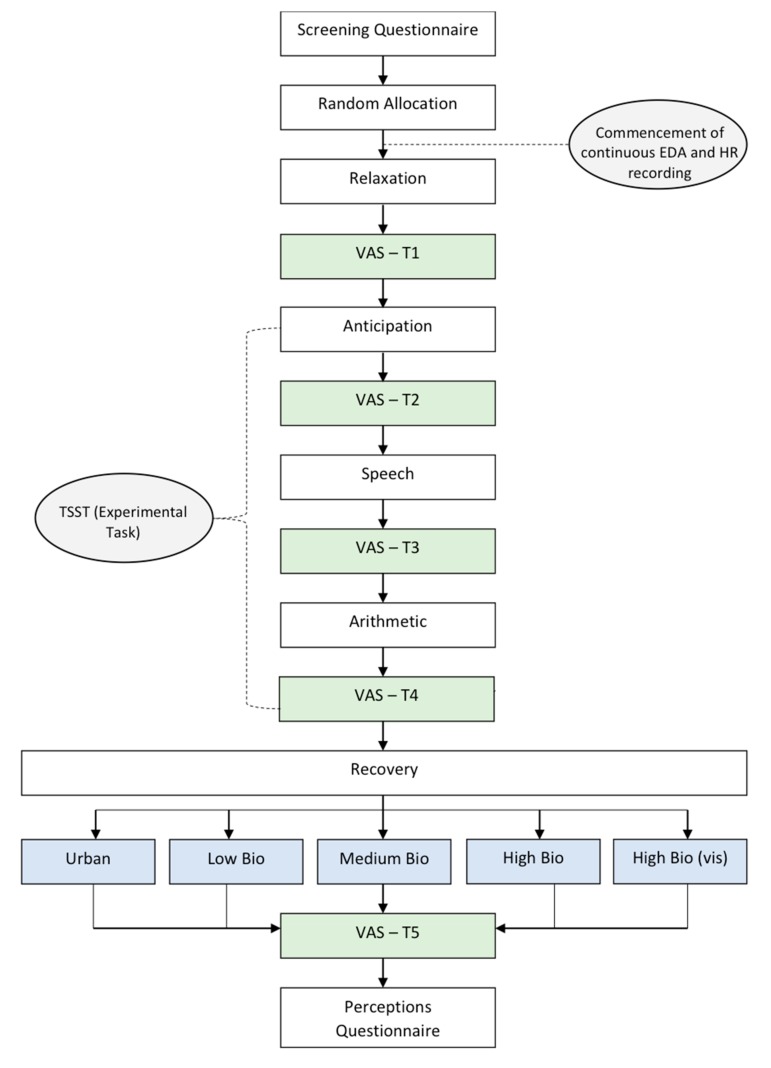
Graphical representation of the IVE experiment procedure. The Visual Analogue Scales (VAS) were completed after each stage of the experiment. Heart rate (HR) and Electrodermal activity (EDA) were recorded continuously throughout the experiment.
